# Work hardening in colloidal crystals

**DOI:** 10.1038/s41586-024-07453-6

**Published:** 2024-05-29

**Authors:** Seongsoo Kim, Ilya Svetlizky, David A. Weitz, Frans Spaepen

**Affiliations:** 1 School of Engineering and Applied Sciences (SEAS), Harvard University, Cambridge, MA USA; 2 Department of Physics, Harvard University, Cambridge, MA USA; 3 Wyss Institute for Biologically Inspired Engineering, Harvard University, Boston, MA USA

**Keywords:** Colloids, Statistical physics, thermodynamics and nonlinear dynamics, Structure of solids and liquids, Metals and alloys

## Abstract

Colloidal crystals exhibit interesting properties^1–4^ that are in many ways analogous to their atomic counterparts. They have the same crystal structures^2,5–7^, undergo the same phase transitions^8–10^, and possess the same crystallographic defects^11–14^. In contrast to these structural properties, the mechanical properties of colloidal crystals are quite different from those of atomic systems. For example, unlike in atomic systems, the elasticity of hard-sphere colloidal crystals is purely entropic^15^; as a result, they are so soft that they can be melted just by stirring^16,17^. Moreover, crystalline materials deform plastically when subjected to increasing shear and become stronger because of the ubiquitous process of work hardening^18^; but this has so far never been observed in colloidal crystals, to our knowledge. Here we show that hard-sphere colloidal crystals exhibit work hardening. Moreover, despite their softness, the shear strength of colloidal crystals can increase and approach the theoretical limit for crystals, a value reached in very few other materials so far. We use confocal microscopy to show that the strength of colloidal crystals increases with dislocation density, and ultimately reaches the classic Taylor scaling behaviour for atomic materials^19–21^, although hard-sphere interactions lack the complexity of atomic interactions. We demonstrate that Taylor hardening arises through the formation of dislocation junctions^22^. The Taylor hardening regime, however, is established only after a transient phase, and it ceases when the colloidal crystals become so hard that the strain is localized within a thin boundary layer in which slip results from an unconventional motion of dislocations. The striking resemblance between colloidal and atomic crystals, despite the many orders of magnitude difference in particle size and shear modulus, demonstrates the universality of work hardening.

## Abstract (web summary)

Deformation of soft colloidal crystals lead to work hardening, similar to that seen in the deformation of metals.

## Main

The properties of atomic crystals under high strain are well established^18^; when subjected to stresses beyond the yield stress, they exhibit plastic flow, which causes an irreversible change in their shape. This is mediated by nucleation and motion of topological line defects called dislocations^18,23^. Increasing plastic deformation requires an increasing flow stress because of the interactions between dislocations. This is work, or strain, hardening. This phenomenon is ubiquitous, yet, owing to the many ways dislocations can interact, our understanding of the mechanism that governs work hardening is still incomplete^20,22,24–26^. In contrast to atomic crystals, colloidal crystals have much simpler interparticle interactions; they consist of solid particles in a fluid and can exhibit purely hard-sphere interactions. Here we show that, despite the simplicity of these interactions, hard-sphere colloidal crystals exhibit work hardening. The micron scale of the particles enables the structure and dynamics of colloidal crystals to be investigated on a particle-by-particle level using optical microscopy^5,8,9,12–14,27–29^. Dislocations in these colloidal crystals can, therefore, be directly visualized in three dimensions and in real time. Thus, these measurements provide insight into the general nature of work hardening.

**Fig. 1:**
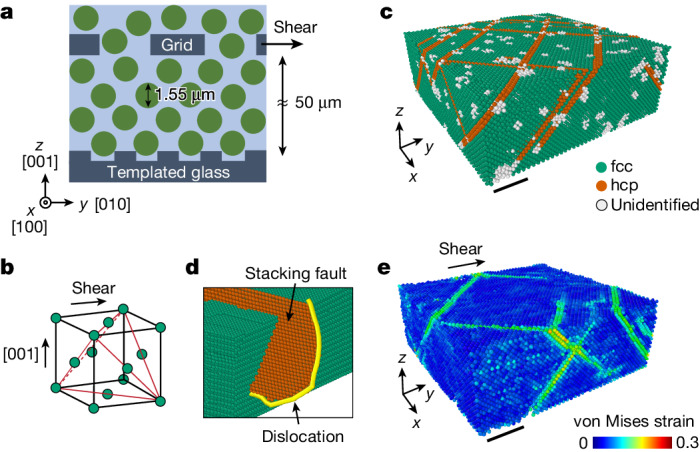
Plastic shear deformation of colloidal single crystals. **a**, Schematic of the experiment. Colloidal crystals are grown by sedimentation of 1.55 μm particles on templates with a square pattern of dimples. The templates dictate the growth of fcc single crystals along the [001] fcc direction. The crystals are sheared by displacing a grid, embedded in the particles, in the [010] fcc direction. **b**, The four close-packed {111} planes are marked in the fcc unit cell. **c**, During the sedimentation process, the hcp stacking faults (orange) in the fcc crystal (green) are formed on the four {111} planes. **d**, The stacking faults arise because of the motion of Shockley partial dislocations (yellow line in **d**) that relax the approximately 1% misfit strain due to the mismatch between the template spacing and the crystal lattice constant. **e**, A snapshot of the von Mises equivalent strain, calculated for *γ* ≈ 0.04 with respect to a reference frame defined at *γ* = 0. Plastic flow is mediated by slip on the {111} fcc planes, the classical easy-glide planes, as shown by the high von Mises strain values (Supplementary Video 5). **c** and **e** correspond to the same region of the crystal. Scale bar, 20 μm (**c**,**e**).

**Extended Data Fig. 1:**
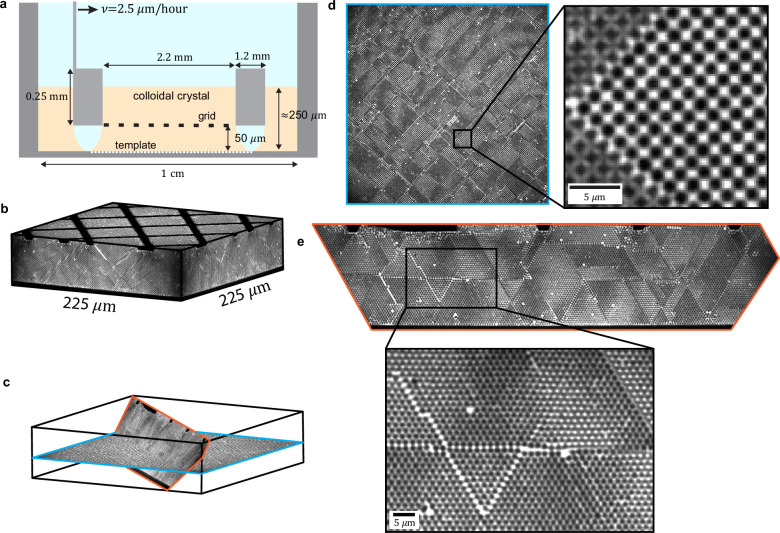
Confocal images of colloidal crystals. **a**, Cross section schematic of the shear cell, not drawn to scale. The colloidal crystals are imaged using a confocal microscope. The volume imaged for one field is 225 *μ*m by 225 *μ*m by 60 *μ*m and contains ~ 1 million particles. **b**, Stack of confocal slices rendered in 3D. **c**, Two slices that are shown in panel **d** and **e**. **d**, A single confocal slice. The particles are black circles because the solvent is dyed. **e**, Confocal images obliquely resliced to show a (111) fcc plane. The stacking faults and dislocations lying on the plane are outlined in side view. Inset: image of a partial dislocation.

We disperse 1.55 μm diameter silica spheres in a mixture of water and dimethyl sulfoxide, which closely matches the refractive index of silica. We dissolve a small amount of fluorescein sodium salt to dye the solvent and additional sodium chloride to further screen the surface charge of the particle to produce a nearly hard-sphere interaction between the particles. The colloidal dispersion is put into a cylindrical shear cell, 1 cm in diameter. The bottom of the cell is a template: a coverslip with a square array of dimples of 1.63 μm spacing (Fig. 1a). The template constrains the first layer of the sedimenting particles and imposes the growth of a face-centred cubic (fcc) single crystal along the [001] fcc direction^30^ (Fig. 1b). We use a spinning-disk confocal microscope to image, at single-particle resolution, five separate regions of 200 × 200 × 60 μm^3^ volume, containing a total of about 5 million particles (Extended Data Fig. 1 and Supplementary Videos 1–3). By processing the confocal images, we locate the position of the particles in three dimensions, which allows us to determine the local crystalline structure^31^ and reconstruct the dislocation lines^32^ with their respective Burgers vectors, **b**.

**Extended Data Fig. 2:**
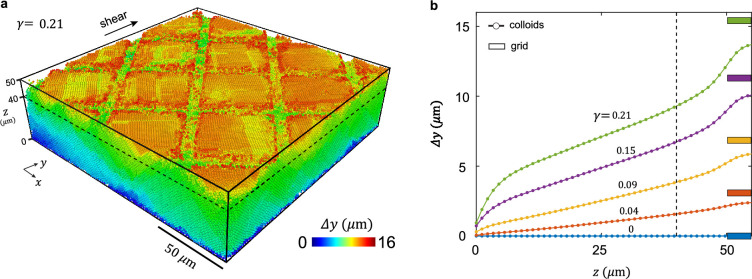
Slip at the top. **a**, A color plot of the measured particle *y*-displacements for *γ=*0.21. The square patterns at the top surface reflect the volume occupied by the grid. **b**, Measured displacement profiles and the imposed displacements of the grid for different values of *γ*. The discrepancies between the measured displacements and the grid position at *z≈*55 *μ*m demonstrate the imperfect coupling between the grid and the crystal. In our current study we ignore this complexity near the grid and focus on particles up to 40 *μ*m (dashed line in panel **a** and **b**) for calculations of *γ* and *γ*_*E*_.

The as-grown fcc crystals contain multiple stacking faults, characterized by local hexagonal close-packed (hcp) stacking, that lie on the {111} planes, as shown by the orange particles in Fig. 1c. Stacking faults are bounded by Shockley partial dislocations (Fig. 1d) and are formed during the growth of the crystal as a result of the approximately 1% misfit strain between the crystal and the template^11^ (Supplementary Video 4). After the particles have fully sedimented, we shear the crystal by displacing a square-meshed grid, embedded 50 μm above the template, as shown schematically in Fig. 1a. The grid moves in the [010] fcc direction at a constant speed of 2.5 μm h^−1^, resulting in a strain rate of 1.4 × 10^−5^ s^−1^ (Methods and Extended Data Fig. 2). During the deformation process, three-dimensional (3D) confocal scans are made every 4 min, fast enough to determine the time-dependent trajectories of the individual particles.

**Extended Data Fig. 3:**
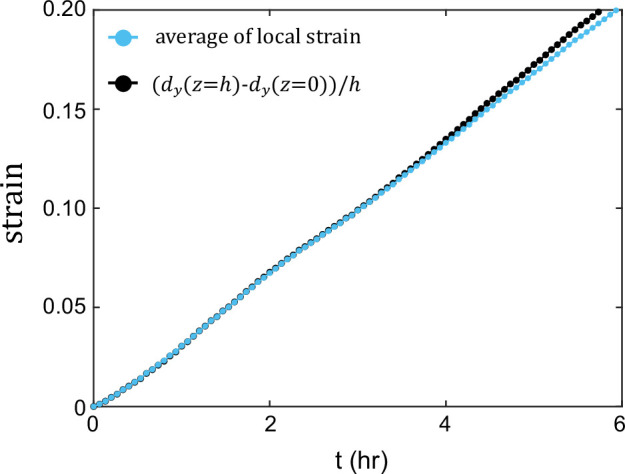
Two ways of measuring strain. For each snapshot, the strain of each particle can be obtained by comparing its nearest neighbor configuration to that prior to the deformation according to ref. ^33^. We take the *yz* component of the resulting strain tensor. The total shear strain (*γ*, cyan) is obtained by averaging over all the particles within at 0<*z<*40 *μ*m. The black line corresponds to the difference of the displacement (*d*_*y*_) between the top and the bottom surface divided by the height *h*, where *h* is 40 *μ*m. The discrepancy between the two measurements is less than 0.005 at all strains.

To quantify the deformation of the sheared crystal, we determine the elastic and total strain fields. The elastic strains are obtained for each snapshot of the particle positions by comparing the local particle configuration with the perfect fcc structure^12,33,34^. By contrast, the total strains are defined with respect to the position of the particles before the deformation^33^, which requires tracking the particle positions in time (Methods). The average elastic (*γ*_E_) and total (*γ*) shear strains are obtained by averaging over all the particles. Here, *γ* is equivalent to the relative displacement of the particles at the top and bottom surfaces divided by its height (Extended Data Fig. 3).

**Fig. 2:**
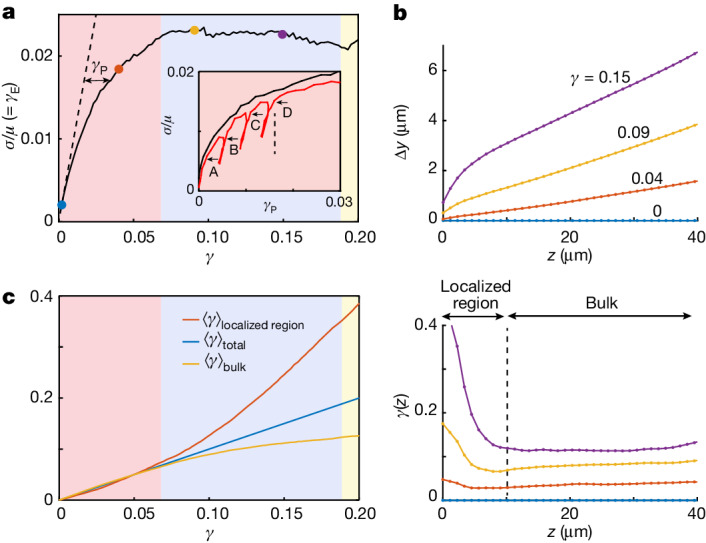
Strain hardening and localization. **a**, The normalized shear stress *σ*/*μ*, where *μ* is the shear modulus, is obtained from the measurements of the elastic strain, *γ*_E_, and plotted as a function of the total strain, *γ*. The dashed line with a slope of unity marks elastic deformation, *γ*_*E*_ = *γ*. The plastic strain, *γ*_P_ = *γ* − *γ*_E_, is denoted by the black double arrow. Inset, strain hardening is demonstrated by replotting *σ*/*μ* as a function of *γ*_P_ (black line); increasing stresses are required to sustain plastic flow. Successive unloading and reloading (red line) demonstrate the irreversibility of the plastic flow; the yield stress, the stress at which the slope d*σ*/d*γ*_P_ suddenly decreases, increases with accumulated *γ*_P_ (A–D). **b**, Profiles of the average particle displacements (top) in the direction of the shear, *y*, binned along the crystal height, *z*, for different values of *γ* (coloured dots in **a**). The corresponding profiles of *γ* (bottom) demonstrate that, at the later stages of the deformation, the strain is localized within a narrow region. **c**, Strains, averaged over the localized region (red line), the bulk region (yellow line) and over the total thickness (blue line), as defined in **b** (bottom). During the strain-hardening regime (red background in **a**,**c**), the crystals are deformed homogeneously. Saturation of the stress marks the onset of a crossover between the homogenous and localized flow regimes (blue background in **a**,**c**). The deformation of the bulk stops entirely at *γ* ≈ 0.2, and the strain is accommodated in the localized region alone (yellow background in **a**,**c**).

We find that the deformation of the crystal is elastic up to *γ* ≈ 0.005; the total strain is accommodated by elastic strain, *γ*_E_ = *γ*, as shown in Fig. 2a by the dashed line with a slope of 1. Beyond this yield point, any further increase in the imposed *γ* causes an increase in the plastic strain, *γ*_P_ = *γ* − *γ*_E_. To determine how *γ*_P_ is accommodated by the crystal, we calculate the spatially resolved von Mises equivalent strain, a scalar invariant that quantifies the maximal shear distortion (Methods). Plastic strain is mediated by slip along the oblique {111} planes, as shown by the high von Mises strain values in Fig. 1e. These measurements show that plastic strain is mediated by the classic fcc slip on the close-packed planes.

The average elastic strains provide a measure of the normalized stresses *σ*/*μ* = *γ*_E_, where *μ* is the shear modulus. Remarkably, our experiments show strain hardening in these hard-sphere colloidal crystals: increasing stresses are required to accommodate plastic flow, as seen by plotting *σ*/*μ* as a function of *γ*_P_, shown by the black line in Fig. 2a (inset). To demonstrate the irreversibility of the plastic flow, we reverse the direction of the shear to remove part of the applied shear stress, and then reload. We find that higher stresses are required to reinitiate plastic deformation: the yield stress, the point at which the slope d*σ*/d*γ*_P_ suddenly decreases, increases with the accumulated *γ*_P_, as denoted by the capital letters (A–D) in Fig. 2a inset. Although the transition to yield is clear, the unloading and reloading stages are not purely elastic, which would require the loading and unloading curves to be strictly vertical (Fig. 2a, dashed line in the inset).

**Extended Data Fig. 4:**
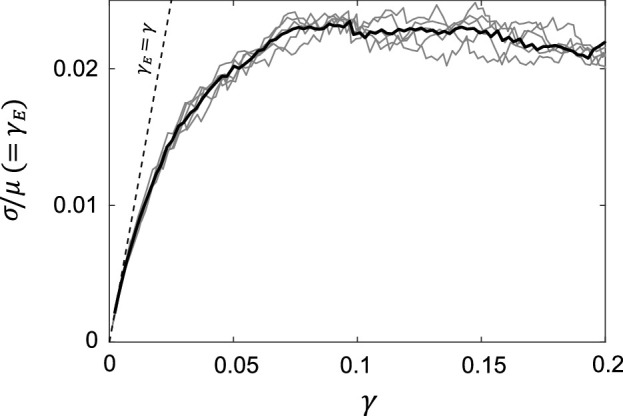
Stress-strain curves from different fields of view. We measure both stress and strain for five fields of view (grey). The average is plotted with a black line.

The strain-hardening stage ends when the stress reaches *σ*/*μ* ≈ 0.02 (Extended Data Fig. 4). Any further flow is maintained at a constant stress level (Fig. 2a). The saturation of stress marks an essential change in the behaviour of the flow. To demonstrate this, we average the particle displacements in the shear direction over the crystalline layers parallel to the substrate. Before the saturation of the stress, the entire crystal undergoes simple shear: the displacement profile is linear, and the strain profile is constant across the thickness of the crystal, as demonstrated by the orange profiles in Fig. 2b. However, during the later stages of the deformation, when the stress saturates, strong displacement gradients near the bottom surface are formed, as shown by the yellow and purple profiles in Fig. 2b (top). The deformation of the crystal is no longer homogeneous, and the strain becomes localized within a 10-μm thick boundary layer at the bottom surface (Fig. 2b, bottom).

To quantify the crossover between the homogenous and localized flow regimes, we average the strain within two separate regions of the crystal: the region in which localization takes place, 0 < *z* < 10 μm, and the bulk of the crystal, in which the deformation is homogenous, 10 μm < *z* < 40 μm. We find that during the strain-hardening stage (red background in Fig. 2a,c), the two strain averages are identical, highlighting again that the deformation is homogeneous over the entire thickness of the crystal. The saturation of the stress, which marks the end of strain hardening, is accompanied by the onset of localization; strain in the localized region exceeds the bulk strain (blue background in Fig. 2a,c). Finally, when the bulk strain saturates, the applied strain is accommodated by the localized region alone (yellow background in Fig. 2a,c).

**Fig. 3:**
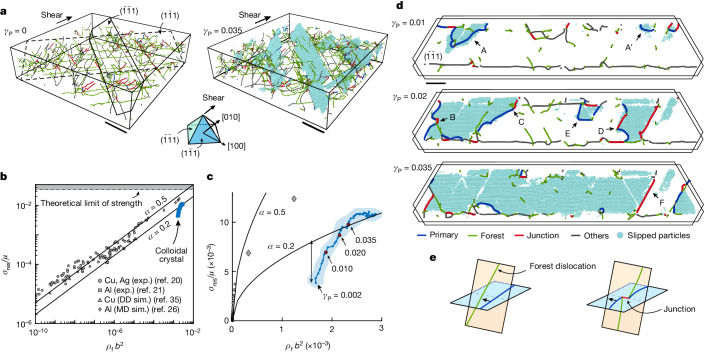
Strain hardening in colloidal crystals. **a**, Snapshots of the dislocation network before (left) and during (right) deformation. The particles displaced by the dominant slip system, ${\bf{b}}=\frac{1}{6}[2\bar{1}1]$ on the $(\bar{1}\bar{1}1)$ plane, are shown in cyan. Another slip system, symmetric with respect to the shear, operates on the $(1\bar{1}1)$ plane (dashed). **b**, Comparisons of the normalized shear stress resolved on the dominant slip systems, *σ*_res_/*μ* (where *μ* is the shear modulus), as a function of the normalized dislocation density, *ρ*_f_*b*^2^ (where *b* is the magnitude of the Burgers vector) for a nearly hard-sphere colloidal crystal and various metallic crystals (Methods). The two solid lines are the predictions of the Taylor equation for two values of *α* (see main text). **c**, Data in **b** are on a linear scale. The double arrow denotes the discrepancy between the Taylor model and the colloidal crystals at the early stages of the deformation. **d**, A 5-μm thick slice of the ($\bar{1}\bar{1}1$) plane (solid line in **a**) is shown for different values of *γ*_P_. At the early stages (*γ*_P_ = 0.01) crystals flow by nucleation of (half) loops of the dominant dislocations (blue segments. Examples are marked by A and A′). At the later stages (*γ*_P_ = 0.02), dislocations can form immobile junctions (red segments) by interaction with the forest dislocations (green segments, see definition in the text), as shown by examples B, C and D. Thereafter, plastic flow is accommodated by unzipping of the junctions (*γ*_P_ = 0.035), although new dislocations can still be formed (E). Some junctions are very strong and limit slip (F). For the complete time series, see Supplementary Video 6. **e**, Schematic showing how dislocations can intersect forest dislocations and form immobile dislocation junctions. Scale bars, 50 μm (**a**); 20 μm (**d**).

**Extended Data Fig. 5:**
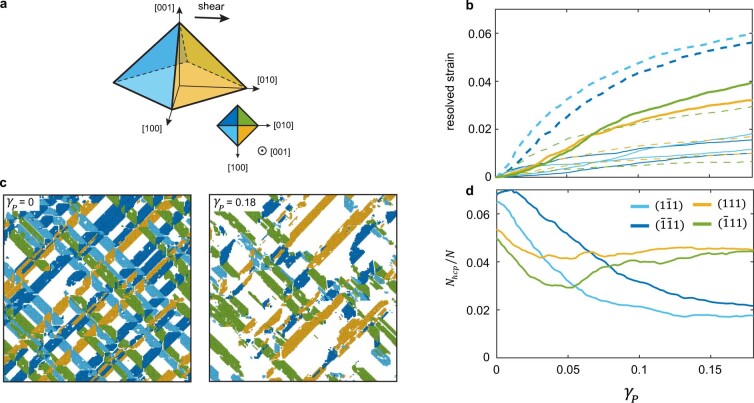
Dominant slip systems and exhaustion of the stacking faults. **a**, A pyramid representation of the four {111} fcc glide planes. **b**, Resolved strains within the bulk for all the slip systems (see legend in Extended Data Table 1). The two dominant slip systems, which are symmetrical with respect to the applied shear, are denoted by blue and cyan dashed lines and correspond to $\vec{b}=\frac{1}{6}[2\bar{1}1]$ and $\frac{1}{6}[21\bar{1}]$ respectively. **c**, Top projection of the stacking faults colored according to their (111) planes prior to the deformation (left) and for *γ*_*P*_ = 0.18 (right). Colors are defined in panel **a**. The crystal is sliced (15 *μ*m < z < 30 *μ*m) for clarity. The blue $(\bar{1}\bar{1}1)$ and cyan $(1\bar{1}1)$ stacking faults are exhausted, leaving behind only small patches of hcp particles. In contrast, the preexisting yellow (111) and green $(\bar{1}11)$ stacking faults are erased and new ones are formed, leaving no substantial changes in their number. **d**, The number of hcp particles on each of the {111} planes, *N*_*hcp*_, normalized by the total number of particles, *N*. The number of hcp particles that correspond to the blue $(\bar{1}\bar{1}1)$ and cyan $(1\bar{1}1)$ planes decreases, reflecting the dominant trailing dislocation (blue and cyan dashed lines) in panel **b**. In contrast, the number of hcp particles that correspond to yellow (111) and green $(\bar{1}11)$ planes remains roughly unchanged, reflecting the balance between the slip mediated by the trailing (dashed lines) and leading (solid lines) dislocation in panel **b**.

To explore the origin of strain hardening, we reconstruct the evolution of the full 3D dislocation network during the deformation process, as shown for *γ*_P_ = 0 (Fig. 3a, left) and *γ*_P_ = 0.035 (Fig. 3a, right). We find that slip is mediated predominantly by two slip systems (Methods and Extended Data Fig. 5): glide of $\frac{1}{6}[2\bar{1}1]$ and $\frac{1}{6}[21\bar{1}]$ Shockley dislocations on, respectively, $(\bar{1}\bar{1}1)$ and $(1\bar{1}1)$ planes (Fig. 3a). The two slip systems are symmetric with respect to the applied shear: we calculate the average the density of all the dislocations that pierce through the two slip planes, which defines the forest dislocation density *ρ*_f_ (Methods). We find that late-stage strain hardening in colloidal crystals is described by the Taylor equation, originally derived to explain strain hardening in atomic crystals^19^: to sustain dislocation glide, the shear stress resolved on a slip system *σ*_res_ (Methods) increases with *ρ*_f_ according to ${\sigma }_{{\rm{r}}{\rm{e}}{\rm{s}}}=\alpha \mu b\sqrt{{\rho }_{{\rm{f}}}}$, where *α* is a dimensionless constant and *b* is the magnitude of the Burgers vector^22^. Moreover, measurements of *σ*_res_/*μ* and *ρ*_f_*b*^2^ in our colloidal crystals (blue symbols) are in accordance with experimental and numerical observations in metallic systems^20,21,26,35^ (grey symbols) and the model predictions (black solid lines for *α* = 0.2 and 0.5) (ref. ^35^), as shown in Fig. 3b. This result is particularly interesting given the four orders of magnitude difference in particle size (0.1 nm to 1 μm) and the nine orders of magnitude difference in the shear modulus (GPa to Pa) between metallic and colloidal crystals. Remarkably, the very dense dislocation network of our colloidal crystals leads to a very high *σ*_res_/*μ* that exceeds that of most metals and ultimately approaches the theoretical limit of strength (Methods). A more detailed investigation of the data shows that the Taylor equation fails to account for the early stages of the strain-hardening regime. The initial dislocation density is the result of a slight tensile mismatch between the crystal and the template; thus, the Taylor equation overestimates the flow stress. Taylor hardening is established only after a transient evolution of the dislocation network (Fig. 3c).

**Extended Data Fig. 6:**
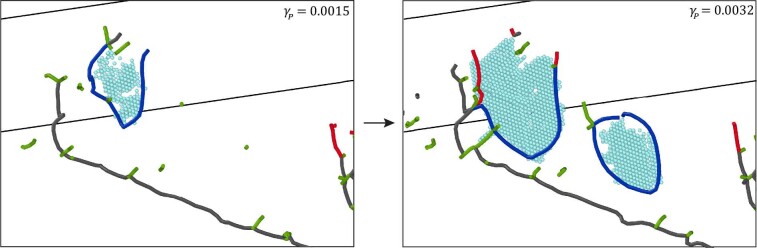
Nucleation of a dislocation loop. Homogenous nucleation of a dislocation loop with the Burgers vector $\frac{1}{6}[21\bar{1}]$ within the crystal. For the color legend, refer to Fig. 3d.

To gain insight into the mechanism of strain hardening, we examine the evolution of slip on a particular $\left(\bar{1}\bar{1}1\right)$ plane, marked by the solid line in Fig. 3a. At early stages of the plastic flow (*γ*_P_ = 0.01), slip is mediated by nucleation of mobile dislocations (blue), as marked by A and A′ in Fig. 3d. We find that nucleation takes place at different parts of the crystal, either by formation of half loops at the vicinity of the upper surface of the crystal (examples A and A′) or by homogeneous nucleation of full dislocation loops within the bulk of the crystal (Extended Data Fig. 6). At the later stages of the flow, dislocations expand (*γ*_P_ = 0.02), and the slipped regions coalesce to cover the entire plane (*γ*_P_ = 0.035), as shown in Fig. 3d.

These observations reveal the underlying mechanism of Taylor hardening. At later stages of strain hardening (*γ*_P_ = 0.02), as the primary dislocations expand, they intersect with Shockley dislocations (Fig. 3e, forest, green) that lie on other glide planes and pierce through the primary plane^22^, as demonstrated by the schematics in Fig. 3e. We provide here direct experimental evidence that interactions between the primary and forest Shockley dislocations result in the formation of sessile junctions. Most of these junctions are of the Lomer–Cottrell type^18^ with ${\bf{b}}=\frac{1}{6}[10\bar{1}]$ (Extended Data Table 2), as shown by the red segments in examples B and D in Fig. 3d. However, we also identify Hirth-type junctions with ${\bf{b}}=\frac{1}{3}[0\bar{1}0]$, as occurs, for example, at C. We find that these dislocation junctions form strong obstacles to the motion of the mobile dislocations, as suggested by the high curvature of the primary dislocations pinned by the junctions^36^ as in example B. Further plastic strain is accommodated by unzipping the immobile junctions (B, C and D). Our measurements show, however, a hierarchy of junction strengths, as some of the junctions remain intact (F), despite the increase in stress. These observations explicitly demonstrate the role of immobile junctions in strain hardening of crystals; to sustain plastic flow, the stress must be sufficiently high to overcome the pinning of dislocations.

Our measurements also show the origin of the discrepancy between the Taylor predictions and the measured flow stress, as observed at the early stages of the strain-hardening regime (Fig. 3c, double arrow). At low strains (*γ*_P_ = 0.01), the dislocations are short and do not interact with the piercing forest dislocations, so that no dislocation junctions have formed yet. Instead, our measurements suggest that the nucleation of dislocations is the dominant mechanism for strain hardening in this case. We find a hierarchy of barriers to dislocation nucleation; although some regions are susceptible to early nucleation at low stress (A and A′), nucleation can also take place at later stages of the flow and at higher stress (E).

**Fig. 4:**
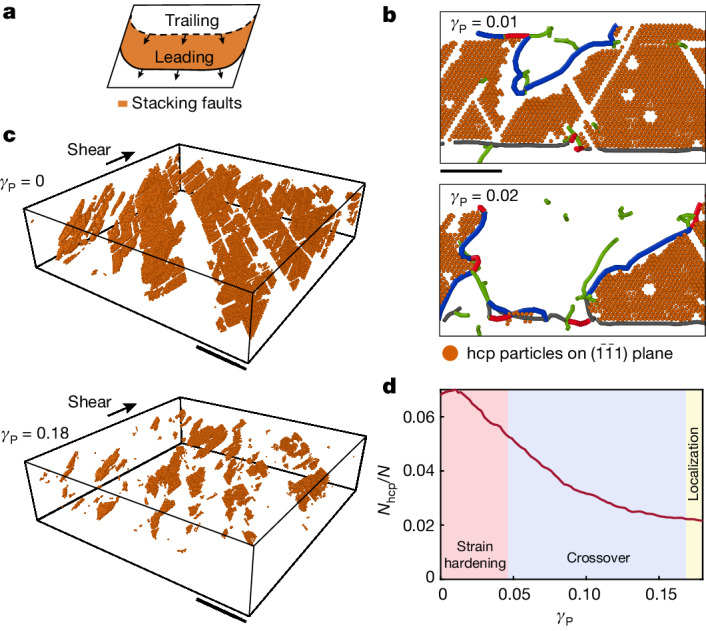
Exhaustion of the stacking faults terminates bulk deformation. **a**, Glide of leading partial dislocations creates hcp stacking faults, whereas glide of trailing dislocations eliminates the stacking faults. **b**, A slice of a $(\bar{1}\bar{1}1)$ plane, corresponding to example A in Fig. 3d. The dominant dislocations (blue) are of the trailing type; $\frac{1}{6}[21\bar{1}]$ dislocations eliminate hcp stacking faults (orange particles) that were left behind by the motion of the Shockley misfit dislocations formed during the epitaxial crystal growth. **c**, Stacking faults that lie on the dominant $(\bar{1}\bar{1}1)$ planes. Stacking faults that were formed before the deformation process (top) are exhausted by the time plastic flow reaches *γ*_P_ = 0.18 (bottom). The crystal was sliced in the range 10 < *z* *<* 40 μm. **d**, Evolution of the number of hcp particles on $(\bar{1}\bar{1}1)$ plane, *N*_hcp_, shown in **c**, normalized by the total number of particles, *N*. The decrease of *N*_hcp_/*N* to small values marks the end of the crossover to localization (blue background). Thereafter, bulk deformation stops entirely (yellow background). Scale bars, 20 μm (**b**); 50 μm (**c**).

Strain hardening in our crystals is interrupted by the onset of localization. We find that the specific geometry of the Shockley partial dislocations limits the strain that can accumulate in the bulk. Unlike perfect dislocations, partial dislocations consist of two types^23^: on applied shear, leading partial dislocations leave behind hcp stacking faults, whereas trailing partial dislocations eliminate the pre-existing stacking faults, as shown in Fig. 4a. We find that the dominant slip systems are of the trailing type. By nucleation and expansion, the $\frac{1}{6}[2\bar{1}1]$ dislocations on the $(\bar{1}\bar{1}1)$ plane negate the pre-existing hcp stacking faults that were left behind by the misfit dislocations during the epitaxial crystal growth^37^ (Fig. 4b). As the plastic flow proceeds, the stacking faults are exhausted, as demonstrated by the number of hcp particles on stacking faults on $(\bar{1}\bar{1}1)$ planes; there is a marked decrease between *γ*_P_ = 0 and 0.18 as shown in the two panels of Fig. 4c. We systematically quantify this process by tracking the fraction of the hcp particles on $(\bar{1}\bar{1}1)$ planes, *N*_hcp_/*N*. These measurements show that the exhaustion of the stacking faults marks the end of the crossover; the deformation of the bulk entirely stops (Fig. 2b, bottom) when the dominant slip systems do not operate because *N*_hcp_/*N* saturates at a low value (Fig. 4d, yellow region).

**Fig. 5:**
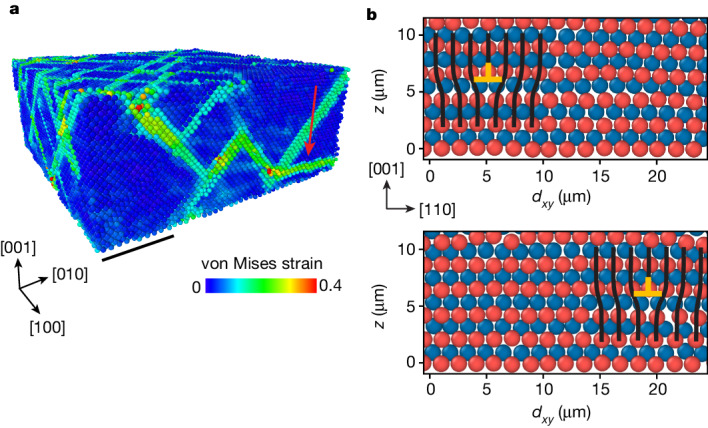
Glide of Lomer dislocations on an unconventional (001) slip plane mediates strain localization. **a**, A snapshot of the von Mises equivalent strain, calculated for *γ* ≈ 0.06, after the onset of the localization of flow. High values show that slip on an unconventional (001) plane has been activated (red arrow), apart from easier slip along the classical {111} planes. **b**, Cross-sections of the colloidal crystal along the $(\bar{1}10)$ plane (see coordinate system in **a**) demonstrate glide of a perfect dislocation ${\bf{b}}=\frac{1}{2}\left[110\right]$ in the [110] direction on a (001) plane. The order of the crystal layers is shown by the red and blue particles, located out of and into the view plane, respectively. Black lines show an extra half-plane. Scale bar, 20 μm (**a**).

Finally, our measurements reveal the mechanism that drives the localization of flow. In contrast to the strain-hardening regime, during which slip occurs along the classic easy-glide {111} planes, flow localization is mediated by slip on an unconventional (001) slip plane, as demonstrated by the red arrow in Fig. 5a. Slip takes place through glide in the $[110]$ and $[1\bar{1}0]$ directions of Lomer dislocations^38–40^, perfect dislocations of edge character in which ${\bf{b}}=\frac{1}{2}[110]$ and $\frac{1}{2}[1\bar{1}0]$ (Fig. 5b). The presence of perfect dislocations in near-hard-sphere colloidal crystal is surprising. Perfect dislocations are expected to dissociate into partial dislocations because of the vanishingly small stacking fault energy. Here, as a result of the applied shear stress, mobile partial dislocations merge with the misfit dislocations to form perfect Lomer dislocations near the bottom interface. Lomer dislocations are often considered to be relatively immobile because of the high frictional forces on the (001) plane^23^. Activation of Lomer dislocations in our experiments is a direct consequence of the severe work hardening of the {111} slip systems, which allows the activation of the less favourable (001) slip system. At this stage, the resolved flow stress is about 0.01*μ* as shown in Fig. 3b; this is close to the theoretical limit of strength and is reached in very few other materials.

We show here that colloidal crystals exhibit work hardening and that their normalized strength approaches the theoretical limit for materials and exceeds that of most atomic systems. The strength of atomic systems is set by the dislocation density; the maximum density is thought to be limited by the annihilation of nearby dislocations^41,42^. The probability of this annihilation process strongly decreases with decreasing stacking fault energy^43,44^. Hard-sphere colloidal crystals have vanishingly small stacking fault energy because of the lack of next-nearest-neighbour interactions. Annihilation of dislocations is not observed in our experiments, which may account for the very high dislocation density and, in turn, very high strength of these crystals. Moreover, we find that, after an initial transient, the relationship between the dislocation density and the strength of the colloidal crystals is in direct agreement with Taylor’s prediction for atomic systems, although hard-sphere interactions lack the complexity of atomic interactions. It is known that the specific details of the interatomic potential can affect, for example, the lattice resistance to the dislocation motion, which, in turn, explains why some atomic crystals break (brittle), whereas others deform plastically (ductile)^18,23^. However, our work demonstrates that colloidal crystals follow the same universal behaviour of many ductile crystals: the exact interparticle interactions and the lattice resistance are of secondary importance to the interactions among dislocations. These measurements provide a new means to study crystal plasticity, as colloidal systems allow unprecedented real-time observation of dislocation dynamics that is inaccessible in atomic crystals. We provide direct experimental evidence that work hardening can be caused by the formation and destruction of sessile dislocation junctions. Although this is widely accepted, it has so far been supported only by numerical simulations^35,45,46^ and indirect experimental evidence^20,47^. Furthermore, the discrepancy between the Taylor equation and the measured flow stress during the transition from the early misfit dislocation configurations to those established by the shear highlights the importance of memory^48^ and suggests that the deformation history is encoded in the structure of the dislocation network. These insights are essential for understanding the classic latent hardening experiments, during which different slip systems are activated successively^47^. Our experiments highlight the competition of the different deformation processes—glide of mobile Shockley and glide of Lomer dislocations—at the limit when the flow stresses approach the theoretical limit of strength. Finally, the exhaustion of stacking faults through the motion of trailing partial dislocations is not unique to colloids but should also be important, for example, in the deformation of nanocrystalline materials^49^, in which the stacking fault and sample sizes are similar. Observation of work hardening in colloidal crystals not only provides insights into this soft matter system but also provides an opportunity to gain important insights into the underlying mechanisms of work hardening itself.

## Methods

### Sample preparation and shear setup

We disperse silica particles (Micromod, Sicastar), 1.55 μm in diameter, in a mixture of dimethyl sulfoxide (DMSO, 62.8% by volume), de-ionized water (36.0% by volume) and fluorescein–NaOH dye solution (1.2% by volume)^50^. The fluorescein–NaOH dye solution is made by dissolving the dye powder (Sigma-Aldrich) into de-ionized water (3% by weight). The final mixture closely matches the refractive index of the particles (*n* = 1.43). We further add 2 mM of sodium chloride to screen the negative surface charge of the particles. Taking into account both the added sodium chloride and the fluorescein–NaOH, we estimate the Debye screening length to be around 6 nm. The particles are washed with several batches of the solvent to obtain reproducible samples.

The shear cell is a stainless steel block with a cylindrical hole 1 cm in diameter and 0.5 cm in depth (Extended Data Fig. 1). The bottom of the cell is sealed with a #1.5 coverslip in which a 5 mm × 5 mm square pattern of wells with 1.63(5) μm spacing has been created by photolithography and reactive ion etching. We attach a mesh grid (spi supplies, G400HS) to a piezoelectric transducer by an extension arm and place it 50 μm above the etched coverslip. We flow into the shear cell a colloidal dispersion with a low volume fraction of about 0.9%. The particles sediment through the grid and form an approximately 60-μm thick crystal after about 6 h. The presence of the etched template dictates the growth of a fcc single crystal with [001] orientation along the *z*-axis. We then add a dose of particles to fully embed the grid inside the crystal, which results in a total thickness of 250 μm. The shear cell is closed by a cap to minimize the evaporation of the solvent and the associated degradation of the image quality. After the particles have fully sedimented, we scan the crystal for another 2 h to verify that the crystal was fully relaxed. The crystal is sheared by displacing the grid at 2.5 μm h^−1^ in the [010] direction of the fcc crystal. The volume fraction of the colloidal crystals, measured both before and during the shear, remains at 68 ± 1%.

### Confocal microscopy

The particles are imaged using a spinning-disk confocal microscope (Yokogawa CSU-W1) with a pinhole 25 μm in diameter. An sCMOS camera (Zyla 4.2) set to 60 ms exposure produces 16-bit images of 1,024 × 1,024 pixels after 2 × 2 binning. The confocal unit is connected to an inverted Leica microscope body (DMi8), with 63× (numerical aperture 1.3) glycerol objective lens, which provides a field of view of 225 μm × 225 μm. We crop a region of 200 μm × 200 μm around the centre to eliminate image aberration. The objective lens is mounted on a piezo-objective scanner (PI, P-725) which allows us to scan a volume of 60 μm in depth with 0.2 μm step size in about 45 s. At this rate, the particles do not move markedly during a single scan. To improve the statistics, we image five different 225 μm × 225 μm fields near the centre of the grid. The volume scans are repeated every 4 min for 12 h. To avoid deterioration of the imaging quality during the experiment, we substitute the Type-G immersion liquid (*n* = 1.45) with a silicone-based liquid, Gelest Alt-143 (Polyoctylmethylsiloxane, *n* = 1.445). The acquired images are processed by commercial deconvolution software (Huygens). The 3D coordinates of the particles are located and tracked using a standard algorithm^51^.

### Calculating the resolved stress and strain for the dominant slip systems

In hard-sphere fcc crystals, slip occurs through the motion of partial dislocations because of the vanishingly small stacking fault energy^52^. Stacking fault energy of hard-sphere crystals is several orders of magnitude smaller than that of atomic crystals^53^. Partial dislocations are restricted to glide on specific crystal planes; in fcc crystals, these are the four close-packed {111} planes. On each of the planes—the so-called slip planes—there are three families of dislocations—the so-called slip systems—distinguished by a unique Burgers vector **b** (Extended Data Table 1). The forces acting on the dislocations depend on the resolved shear stresses *σ*_res_—the stress projected on the slip plane and in the slip direction **b**. *σ*_res_ is related to the applied stress *σ* through the geometrical Schmid factor *S* by *σ*_res_ = *Sσ*. For an applied shear stress in the [010] direction (the *yz* shear in Fig. 1a) *S* = |*b*_*y*_*n*_*z*_ + *b*_*z*_*n*_*y*_|/|**b**||**n**|, where **n** is the vector normal to the slip plane^23^. Four of the slip systems, one on each of the four planes, are characterized by *S* = 0.47, whereas for the remaining eight slip systems *S* = 0.23. The 12 slip systems with their **n**, **b** and *S* are summarized in Extended Data Table 1.

Although usually slip systems with the largest Schmid factor are considered the dominant slip systems, recent work showed that this is not always the case^26^. Therefore, we identify the dominant slip systems by directly measuring the resolved strain for each slip system as follows. We first identify the slipping particles. We compute the deformation gradient tensor *α*_*ij*_ and the total strain tensor *ε*_*ij*_ by following the methods described in ref. ^33^. To this end, we define the neighbouring particles according to a cutoff radius *r*_c_ = 3 μm, a value slightly larger than the position of the first minimum of the radial distribution function (about 2 μm). This choice reduces the noise in the strain measurements that originates from the particle locating error. From the strain tensor, we calculate the von Mises equivalent strain ${\varepsilon }_{{\rm{vM}}}={\left[{\varepsilon }_{xy}^{2}+{\varepsilon }_{xz}^{2}+{\varepsilon }_{yz}^{2}+\frac{1}{6}\{{({\varepsilon }_{xx}-{\varepsilon }_{yy})}^{2}+{({\varepsilon }_{xx}-{\varepsilon }_{zz})}^{2}+{({\varepsilon }_{yy}-{\varepsilon }_{zz})}^{2}\}\right]}^{0.5}$, which quantifies the deviatoric (shear) part of the strain tensor. Particles that have slipped have high von Mises strain values, as can be seen in Fig. 1e. We, therefore, identify the slipping particles between two consecutive frames as those with *ε*_vM_ > 0.03. This threshold value depends on *r*_c_ and was found to accurately trace the movement of dislocations^54^.

Next, we classify the slipping particles according to one of the 12 slip systems *s* = 1, 2, 3, … 12. We compare the measured *α*_*ij*_ with the deformation gradient tensor due to the motion of a dislocation, ${F}_{ij}^{s}=\frac{b\delta A}{V}({\hat{b}}_{i}{\hat{n}}_{j})$, where *V* is the volume, **b** is the Burgers vector, *δA* is the slipped area, and **n** is the normal vector of the glide plane that corresponds to *s*. In practice, *s* is determined for each particle by minimization of ${D}^{s}=\sum _{i,{j}}{({\alpha }_{{ij}}-{{qF}}_{{ij}}^{s})}^{2}$ with respect to the scalar *q*. The resolved plastic strain for slip system *s* is ${\varepsilon }_{ij}^{P}=\frac{b\delta A}{2V}({\hat{b}}_{i}{\hat{n}}_{j}+{\hat{b}}_{j}{\hat{n}}_{i})$. The slipped area is $\delta A=\frac{{N}_{s}}{2}\frac{{a}^{2}\sqrt{3}}{4}$, where *a* is the lattice constant (*a* = 2.26 μm), and *N*_*s*_ is the number of slipping particles in slip system *s*. Here we include only the bulk region (see definition of bulk in Fig. 2b), where slip takes place on the {111} planes. This analysis shows that there are two dominant slip systems, ${\bf{b}}=\frac{1}{6}[2\bar{1}1]$ and $\frac{1}{6}[21\bar{1}]$, which are symmetric with respect to the imposed shear and contribute about 40% of the total plastic strain (Extended Data Fig. 5b). In Fig. 3b, we consider these two slip systems and calculate *σ*_res_ by using *S* = 0.47.

### Dislocation density measurements

A simple way to measure the dislocation density in the bulk would be to divide the total length of all the dislocation segments *L* by the crystal volume, *V*. This is referred to as the total dislocation density *ρ*_T_. However, the motion of the specific dislocation family—slip system—is hindered by all the dislocations that pierce the specific slip planes—the dislocation forest^20,45^. We calculate the forest dislocation density in the bulk *ρ*_f_ for the specific slip system by taking the average of the total number of piercing dislocations per area over all the parallel crystallographic planes. We take the average of *ρ*_f_ over the two dominant slip systems as these are symmetric with respect to the applied shear. We find that *ρ*_f_/*ρ*_T_ ≈ 0.4, which is consistent with numerical simulations^45^. For the strain-hardening analysis in Fig. 3b,c, we normalize *ρ*_f_ by the magnitude of the Burgers vector *b* of the two dominant slip systems, which is measured to be 0.92 μm.

### Metal data

All the experimental metal data for the resolved shear strength and dislocation density in Fig. 3b were obtained by performing uniaxial tensile deformation tests^20,21^. The molecular dynamics simulation data were obtained from the uniaxial tensile tests of aluminium along the [001] crystal axis^26^. The reported tensile stresses and dislocation densities at five strains (0.025, 0.200, 0.400, 0.600 and 0.77) are normalized with the Burgers vector and the shear modulus of aluminium; the resolved shear stresses are calculated with a Schmid factor of 0.4082, which remains constant throughout the deformation. Forest dislocation densities (the densities of the piercing dislocations from the other three slip planes) are taken to be three-quarters of the total dislocation densities^35^.

### Theoretical limit of strength

The theoretical limit of strength is the stress required to plastically deform an ideal—infinite, defect-free—crystal by simultaneously displacing all atoms across two crystal planes separated by a distance *h*. In the simplest case^55^, the shear stress *σ* varies periodically with the lattice constant *b* and has a sinusoidal functional form with the shear displacement *x*. The maximum value of *σ*(*x*) = *σ*_max_ = *μb*/2π*h* is the theoretical limit of strength. In a more realistic case in which the fcc crystal structure is considered, the strength *σ*_max_ ≈ 0.033*μ* (ref. ^56^) (Fig. 3b, dashed line). This limit serves as the upper bound to the strength of real materials as it neglects the presence of dislocations that provide a much more efficient mechanism for plastic deformation.

### Physical interpretation of *α*

In the Taylor equation ${\sigma }_{{\rm{res}}}=\alpha \mu b\sqrt{{\rho }_{{\rm{f}}}}$, the non-dimensional numerical constant *α* incorporates the details of the dislocation interactions. In the forest hardening model, primary dislocations are pinned by forming two immobile dislocation junctions on each side; the linear elastic framework predicts that the stress needed to break the junctions is proportional to *μb*/*l*, where *l* is the distance between the two junctions and is related to the dislocation density *ρ*_f_ by *l* = 1/√*ρ*_f_. The proportionality factor depends on the incident angle between the primary and forest dislocations, the character of the dislocations (screw or edge) and the type of the formed junction (Lomer–Cottrell, Lomer, Hirth, Collinear and others). The proportionality factor *α* can be calculated, in principle, by a proper averaging of all these ingredients. In fcc crystals, the theoretical^22,57^, experimental^20^ and numerical^35^ estimates of *α* fall in the range of 0.2–0.5. Dislocation dynamics simulations, which allow the examination of a single type of dislocation junction, estimate *α* ≈ 0.7 for collinear interactions, and *α* ≈ 0.2 and 0.3 for Hirth and Lomer–Cottrell dislocations, respectively^58^. The good agreement of our measurements with the lower bound (*α* = 0.2) can be explained by our observations that in colloidal crystals, the formed dislocation junctions are mostly of Lomer–Cottrell and Hirth type.

## Online content

Any methods, additional references, Nature Portfolio reporting summaries, source data, extended data, supplementary information, acknowledgements, peer review information; details of author contributions and competing interests; and statements of data and code availability are available at 10.1038/s41586-024-07453-6.

### Supplementary information

Supplementary Video 1 A stack of confocal microscope images showing a single partial dislocation, at the end of a stacking fault in a colloidal crystal.

Supplementary Video 2 A stack of confocal microscope images showing a typical colloidal crystal before shear. Lines that seem to separate different parts of the crystals correspond to stacking faults.

Supplementary Video 3 Confocal images in the middle of the colloidal crystal during shear.

Supplementary Video 4 Three-dimensional representation of the dislocation network before shear.

Supplementary Video 5 Rendered images of a colloidal crystal with its local von Mises strains during shear up to *γ* = 0.12.

Supplementary Video 6 Dislocation dynamics of the dominant slip system during shear. Dislocations nucleate, expand and form junctions by interacting with the misfit dislocations that act as forest dislocations.

## Figures and Tables

**Extended Data Table 1:**
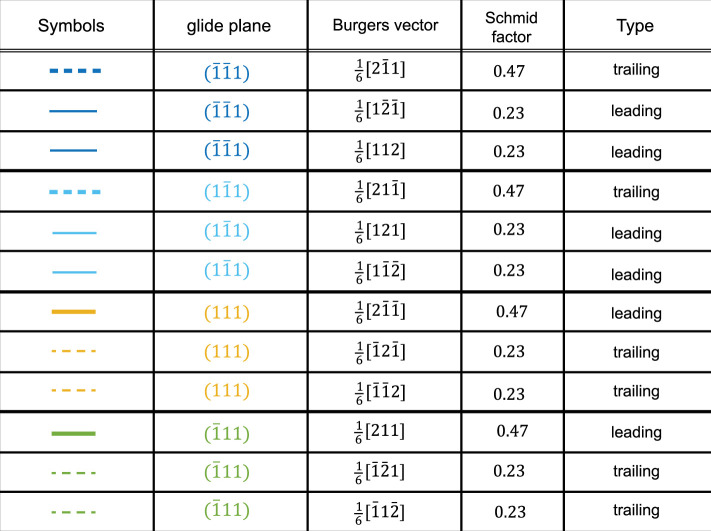
FCC slip systems

A list of all partial dislocation slip systems with the corresponding glid planes, Burgers vectors, Schmid factors and types (leading and trailing dislocation types are defined in the main text). Burgers vectors are in units of the fcc lattice constant (*α*). Schmid factors are computed for simple shear along [010].

**Extended Data Table 2:**
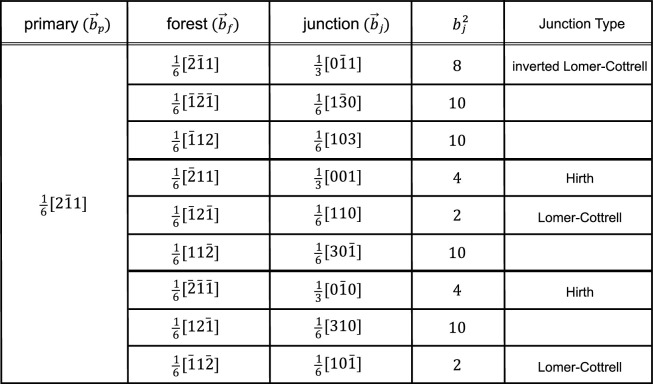
Types of dislocation junctions that can be formed in colloidal crystals

When a primary partial dislocation with the Burgers vector ${\vec{b}}_{p}$ interact with a forest dislocation with ${\vec{b}}_{f}$, they form a dislocation junction with the Burgers vector ${\vec{b}}_{j}$ where ${\vec{b}}_{j}={\vec{b}}_{p}+{\vec{b}}_{f}$. The Lomer-Cottrell dislocation junctions are considered to be an effective junction^23^ because of its low elastic energy per unit length, which is proportional to ${b}_{j}^{2}$. Burgers vectors are in units of the fcc lattice constant (*a*). ${b}_{j}^{2}$: in units of *α*^2^/36.

## Data Availability

The data that support the findings of the study are available from the corresponding author.
